# 

**Published:** 1881-03

**Authors:** 


### BOOKS AND PAMPHLETS RECEIVED.

Excision of Cancer of the Rectum, a Study of 140 Cases. By Chas. B. Kelsey, M. D.

Report of the Louisiana State Board of Health for 1880.

On the Introduction of Food and Medicine into the Stomach when the Ordinary Channel is obstructed. By T. Humbert, M. D.

Electricity in Medicine and Surgery. By John J. Caldwell, M. D.

Errors in the Diagnosis of Eye Diseases. By S. Theobald, M. D.

Catalogue of Medical School, Harvard University, 1880-81.

The Surgical Treatment of Cancer of the Rectum. By Chas. B. Kelsey, M. D.

The Sanitary Problems of Chicago, Past and Present. By John H. Rauch, M. D., Secretary State Board of Health of Illinois.

The Development of the Osseous Callus in Fractures of the Bones of Man and Animals. By H. O. Marcy, M. D.

The Treatment of Ophthalmia, Purulenta Gonorrhorica and Neonatorum, with Clinical Remarks. By C. A. Lambert, M. D.

Abdominal Pulsation, Simulating Aneurism of the Abdominal Aorta. By John Williams, M. D.

Anæmia in Infancy and Early Childhood. By A. Jacobi, M. D.

Cæsarean Section (Porro-Müeller method) with Removal of Uterus and Ovaries. By Elliott Richardson, M. D.

The Treatment of Hip Disease. By E. H. Bradford, M. D.

Winter Health Resorts. By Boardman Reed, M. D.

Scarlatina. By Dr. Wm. B. Atkinson.

Cases Treated by the Lister Method. By T. H. Gerrish, M. D.

Phthisis Pulmonalis. By L. De Brémon, M. D.

Otitus Medica Non—Suppurativa Chronica—1,000 Cases. By E. E. Holt, M. D.

The Asylums of Europe. By Geo. M. Beard, M. D.

Minor Surgical Gynæcology. By Paul F. Munde, M. D.

Transactions of the Minnesota State Medical Society for 1880.

Bulletins of the Public Health. Issued by the Supervising Surgeon General, Washington, D. C., 1880.

Proceedings Louisiana State Medical Association for 1880.

Hand Book of Urinary Analysis, Chemical and Microscopical. By F. M. Deems, M. D.

Second Biennial Report of the Illinois Eastern Hospital for the Insane, at Kankakee, Ills. October 1, 1880.

Report of Albany Hospital for two years ending January 31, 1880.

Rocky Mountain Health Resorts. By Chas. Dennison, M. D.

Transactions State Medical Society of Wisconsin for 1880.

Atlas of Histology. By E. Klein, M. D., and E. N. Smith, M. D. Parts xii and xiii.

Atlas of Skin Diseases. By L. A. Duhring, M. D. Part vii.

---

SPECIAL NOTICE.—We desire to inform the friends of the JOURNAL AND EXAMINER, that a man by the name of W. G. Gano, is going about the country collecting money on the JOURNAL account and making no return of it here. He has *no authority to make such collections.*

The JOURNAL employs no agents. It endeavors to transact all of its business *directly* with the profession. We take this method of informing the profession that, in paying money to any person representing himself as our agent, they do it at their own risk.

WHOOPING COUGH has been successfully treated by Dr. Barety, of Nice, by turpentine vapor. By accident, a child severely affected, was allowed to sleep in a room, recently painted and redolent with turpentine odor, when noticeable improvement took place. Dr. B. has since employed this drug, placed in plates and allowed to stand in the rooms occupied by whooping cough patients. He holds that the disease is mitigated and its duration lessened by this simple expedient.

### SOCIETY MEETINGS.

Chicago Medical Society—Mondays, March 7 and 21.
West Chicago Medical Society—Mondays, March 14 and 28.
Biological Society—Wednesday, March 2.

### CLINICS.

MONDAY.

Eye and Ear Infirmary—2 p. m., Ophthalmological, by Prof. Holmes; 3 p. m., Otological, by Prof. Jones.

Mercy Hospital—2 p. m., Surgical, by Prof. Andrews.

Rush Medical College—2 p. m., Dermatological and Venereal, by Prof. Hyde.

Woman's Medical College—2 p. m., Dermatological and Venereal, by Prof. Maynard; 3 p. m., Diseases of the Chest, Prof. Ingals.

TUESDAY.

Cook County Hospital—2 to 4 p. m., Medical and Surgical Clinics.

Mercy Hospital—2 p. m., Medical, by Prof. Quine.

WEDNESDAY.

Chicago Medical College—2 p. m., Eye and Ear, by Prof. Jones.

Rush Medical College—2 p. m., Medical, by Dr. Bridge; 3 p. m., Ophthalmological and Otological, by Prof. Holmes; 3:30 to 4:30 p. m., Diseases of the Chest, by Dr. E. Fletcher Ingals.

THURSDAY.

Chicago Medical College—2 p. m., Gynæcological, by Prof. Jenks.

Rush Medical College—2 p. m., Diseases of Children, by Dr. Knox; 3 p. m., Diseases of the Nervous System, by Prof. Lyman.

Eye and Ear Infirmary—2 p. m., Ophthalmological, by Dr. Hotz.

Woman's Medical College—3 p. m., Surgical, by Prof. Owens.

FRIDAY.

Cook County Hospital—2 to 4 p. m., Medical and Surgical Clinics.

Mercy Hospital—2 p. m., Medical, by Prof. Davis.

SATURDAY.

Rush Medical College—2 p. m., Surgical, by Prof. Gunn; 3 p. m., Orthopœdic, by Prof. Owens.

Chicago Medical College—2 p. m., Surgical, by Prof. Isham; 3 p. m., Neurological, by Prof. Jewell.

Woman's Medical College—11 a. m., Ophthalmological, by Prof. Montgomery; 2 p. m., Gynæcological, by Prof. Fitch.

Daily Clinics, from 2 to 4 p. m., at the Central Free Dispensary, and at the South Side Dispensary.

